# Psychosocial and Occupational Influences on Self-Induced Hypoglycemia in Type 1 Diabetes

**DOI:** 10.1192/j.eurpsy.2026.11316

**Published:** 2026-07-31

**Authors:** N. Gannoun, F. Ben Abdesslem, G. Saad, I. Halloul, N. Belhadj, F. Chelly, C. Sridi, A. Fki, M. Maoua, Y. Hasni

**Affiliations:** 1 Occupational Medicine Department, Sahloul University Hospital; 2 Faculty of Medicine of Sousse, University of Sousse; 3 Sousse, Research laboratory (LR19SP03); 4 Department of Endocrinology, University Hospital of Farhat Hached Sousse, Sousse, Tunisia

## Abstract

**Introduction:**

Factitious hypoglycemia represents a diagnostic challenge in endocrinology, particularly among patients with type 1 diabetes. Beyond metabolic abnormalities, such cases often reflect underlying psychiatric disorders and maladaptive coping strategies. More broadly, psychosocial stressors- such as occupational strain, interpersonal conflict, or perceived discrimination- are increasingly recognized as contributors to both the onset and perpetuation of self-harming behaviors in vulnerable individuals.

**Objectives:**

To highlight the interplay between psychiatric morbidity, occupational stressors, and maladaptive coping in the occurrence of factitious hypoglycemia, and to underline the need for integrated psychosocial assessment in atypical endocrine presentations.

**Methods:**

We conducted a descriptive case analysis integrating clinical, biochemical, and psychosocial findings, complemented by psychiatric evaluation.

**Results:**

We report the case of a 29-year-old woman with type 1 diabetes since early childhood, also treated for autoimmune hypothyroidism, who was admitted for recurrent severe hypoglycemia (blood glucose as low as 0.26 g/L). She reported persistent hypoglycemia despite discontinuing insulin therapy for one month. Clinical examination was unremarkable. On further inquiry, she admitted to intentional self-injection of rapid-acting insulin, within a broader context of chronic occupational stress, strained hierarchical relations, and a strong perception of gender-based discrimination in the workplace. Psychiatric assessment confirmed a major depressive episode, leading to the initiation of antidepressant therapy and referral for specialized psychiatric follow-up.

**Conclusions:**

**Discussion and Conclusion:** This case illustrates how the workplace environment can profoundly influence the psychological well-being of individuals living with chronic diseases, who may be both physically and emotionally vulnerable. In such contexts, maladaptive coping behaviors, including factitious hypoglycemia, may emerge as a response to overwhelming stress or perceived injustice. Recognizing these patterns requires clinicians to look beyond biochemical findings and to integrate the psychosocial dimension into their assessment. For endocrinologists, the identification of such atypical cases should prompt close collaboration with psychiatry and occupational medicine, ensuring a coordinated, multidisciplinary approach that addresses both medical stability and psychological resilience, while also supporting healthier professional integration.

**Disclosure of Interest:**

None Declared

